# Targeting Apolipoprotein C-III for the Management of Severe Hypertriglyceridemia: Current Research and Future Directions

**DOI:** 10.7759/cureus.67091

**Published:** 2024-08-17

**Authors:** Mili Shah, Abisheikh Sharma, Mohammed Ayyad, Ethan Swartz, Danyaal Jafrani, Dhir Gala

**Affiliations:** 1 Internal Medicine, American University of the Caribbean School of Medicine, Sint Maarten, SXM; 2 Internal Medicine, Rutgers University New Jersey Medical School, Newark, USA

**Keywords:** cardiovascular risk, volanesorsen, antisense oligonucleotides, triglyceride regulation, genetic variations, lipid metabolism, apolipoprotein c-iii, hypertriglyceridemia

## Abstract

Hypertriglyceridemia is characterized by elevated triglyceride levels in the blood, which increases the risk of cardiovascular disease and pancreatitis. This condition stems from multiple factors including lifestyle choices, genetics, and conditions such as diabetes and metabolic syndrome. Apolipoprotein C-III (APOC3), a protein for lipid metabolism, hinders enzymes necessary for breaking down triglycerides and thus plays a key role in hypertriglyceridemia. Variations in the APOC3 gene are associated with varying triglyceride levels among individuals. Recent genetic studies and clinical trials have shed light on the potential of targeting APOC3 as a potentially promising therapeutic modality of hypertriglyceridemia. Antisense oligonucleotides like volanesorsen have displayed effectiveness in lowering triglyceride levels in individuals with severe hypertriglyceridemia. This review article delves into how APOC3 influences triglyceride control and its potential use in targeting APOC3 to manage severe hypertriglyceridemia.

## Introduction and background

Hypertriglyceridemia is a condition marked by elevated levels of triglycerides in the blood. These triglycerides can increase the risk of cardiovascular disease and pancreatitis [1]. Hypertriglyceridemia is categorized as mild to moderate when levels are greater than 150 mg/dL-499 mg/dL, whereas severe hypertriglyceridemia is categorized by levels surpassing 500 mg/dL [2].

Hypertriglyceridemia can be caused by factors such as genetics, lifestyle choices including diet and physical inactivity, and underlying comorbidities like diabetes and metabolic syndrome [3]. Understanding how triglyceride levels are regulated is crucial for devising treatments for this condition.

Apolipoprotein C-III (APOC3) is a protein that plays an important role in lipid metabolism. Mainly produced in the liver with some contribution from the intestines, APOC3 is found in very low-density lipoproteins (VLDLs) and high-density lipoproteins (HDLs) [4]. It’s known to inhibit the activity of enzymes such as lipoprotein lipase (LPL) and hepatic lipase, which are crucial for breaking down triglycerides [5]. The APOC3 gene resides on chromosome 11, where it encodes the APOC3 protein [6]. Variations in the APOC3 gene can result in variations in triglyceride levels among individuals. Specifically, certain changes or differences in the APOC3 gene have been linked to different levels of triglycerides [7].

This review of the existing literature aimed to offer insight into how APOC3 impacts adults with hypertriglyceridemia. By examining research on APOC3, the goal was to uncover how this gene influences triglyceride levels and investigate potential treatment implications by targeting APOC3 for severe hypertriglyceridemia management.

## Review

Pathophysiology of hypertriglyceridemia

The incidence of hypertriglyceridemia rises with increasing age and is influenced by both environmental and genetic factors [8]. In the United States, around 25% of adults have triglyceride levels exceeding 150 mg/dL [9]. Hypertriglyceridemia can stem from primary or secondary causes. Primary causes are usually linked to genetic mutations impacting lipid metabolism leading to disorders such as familial combined hyperlipidemia. This condition is caused by defective mutations involving enzymes and proteins involved in metabolism, such as LPL and APOC3 [10].

Secondary causes that can contribute to overall triglyceride levels involve acquired conditions or lifestyle choices. Common secondary factors that contribute to hypertriglyceridemia include obesity, metabolic syndrome, diabetes, alcohol consumption, medications, and comorbidities [11]. Conditions such as obesity and metabolic syndrome are closely linked to triglyceride levels due to excess lipid buildup and insulin resistance, which increases the likelihood of developing atherosclerotic plaques [12,13]. Managed diabetes, especially type 2 diabetes, is often associated with increased triglycerides [14]. Eating a diet high in carbs, sugars, and saturated fats can also raise levels as can consuming excessive amounts of alcohol [15]. Certain medications such as corticosteroids, beta-blockers, and estrogens have been known to elevate triglycerides [16]. In addition, medical issues such as hypothyroidism, chronic kidney disease, and specific autoimmune disorders may also play a role in raising triglyceride levels [17,18].

While hypertriglyceridemia is often asymptomatic, it can present with signs and symptoms when triglyceride levels are significantly elevated. Some notable clinical features include xanthomas, lipemia retinalis, and acute pancreatitis. High levels of triglycerides also pose cardiovascular-related risks including atherosclerosis build-up, myocardial infarction, and stroke [19]. Understanding the underlying process of hypertriglyceridemia is crucial for developing treatments. APOC3 plays a role in regulating metabolism offering valuable insights into the conditions, mechanisms, and potential treatment targets.

Role of APOC3 in lipid metabolism

APOC3 plays an integral role in managing lipid levels in the body. It is mainly found in the liver and to a certain extent in the intestines. APOC3 is a component of VLDLs and HDLs. One of the functions of APOC3 is to inhibit the activity of LPL and hepatic lipase enzymes, which are responsible for breaking down triglycerides. By inhibiting these enzymes, APOC3 helps regulate the breakdown and the release of free fatty acids, ultimately controlling lipid levels in the blood [20,21].

The regulation of LPL and hepatic lipase by APOC3 is vital for maintaining lipid balance. LPL is present on the endothelial surfaces of tissues like muscles and fat and aids in breaking down triglycerides into free fatty acids and glycerol from chylomicrons and VLDLs for tissue absorption [22]. Hepatic lipase, predominantly found in the liver, also assists in breaking down triglycerides from lipoproteins into particles that can be removed from circulation [23,24]. By inhibiting these enzymes, APOC3 slows down the removal of lipoproteins, leading to increased plasma triglyceride levels.

APOC3 plays a role in more than just lipid processing; it is also linked to metabolic disorders. When APOC3 levels are high, the likelihood of developing metabolic syndrome and type 2 diabetes increases. This is because APOC3 not only influences lipid metabolism but also impacts insulin sensitivity. Elevated APOC3 levels can worsen insulin resistance, triggering a cycle that elevates triglyceride levels and raises the risk of metabolic syndrome and diabetes. The connection between APOC3 and insulin resistance is complex and significant [25,26]. Insulin resistance occurs when muscle, fat, and liver cells become less responsive to insulin, which decreases their capacity to absorb glucose from the blood, resulting in high blood sugar levels. Various studies have revealed that increased APOC3 levels can exacerbate insulin resistance. Studies have demonstrated that APOC3 can directly inhibit insulin signaling pathways. Previous research has shown that APOC3 expression in mice led to impaired liver insulin signaling, causing reduced glucose tolerance and heightened insulin resistance [27-29]. This indicates that aside from its impact on lipid metabolism, APOC3 also directly influences glucose metabolism.

Studies on variations related to APOC3 have shed light on its significance in managing metabolism. Some genetic mutations in the APOC3 gene have been linked to lower levels of triglycerides and a decreased risk of cardiovascular morbidity and mortality. These gene mutations often lead to variants that reduce the production of APOC3, a protein that inhibits the breakdown of fats in the blood and boosts the creation of VLDLs. This results in improved lipid processing and lower VLDL levels among individuals with these genetic changes, which contributes to lower lipid levels. Studies show that people carrying these mutations not only have healthier triglyceride levels but also face fewer cardiac-related issues and have reduced cardiovascular mortality rates [30,31]. These discoveries have sparked interest in creating treatments that mimic the effects of these mutations, one of the most notable ones being antisense oligonucleotides that target APOC3 mRNA, aiming to lower the production of APOC3 protein, which is associated with higher triglyceride levels [32,33].

In essence, APOC3 plays a role in regulating metabolism and significantly impacts blood triglyceride levels. Its function in hindering lipase activity and encouraging VLDL secretion highlights its significance in the development of hypertriglyceridemia. Understanding how APOC3 functions provides possibilities for treatments that aim to reduce levels and lessen the risks associated with heart and metabolic diseases [4,20,34].

APOC3 and severe hypertriglyceridemia

The impact of APOC3 on hypertriglyceridemia has been extensively studied. Several research studies have emphasized the role of APOC3 in the development and progression of severe hypertriglyceridemia, providing valuable insights into the underlying mechanisms and possible treatment targets [30,34,35]. A study published in the New England Journal of Medicine investigated how different variations in the APOC3 gene affect levels and cardiovascular risks. The researchers found that individuals with loss-of-function mutations in the APOC3 gene had notably lower triglyceride levels and a significantly decreased occurrence of ischemic heart disease compared to those without such mutations. This study presented evidence that reducing APOC3 activity has a protective impact on heart disease [31].

Clinical trials have also delved into targeting APOC3 for treating hypertriglyceridemia. One promising method involves using antisense oligonucleotides to block APOC3 expression. Antisense oligonucleotides are artificial strands of genetic material designed to specifically attach to the mRNA of a target gene like APOC3 [36,37]. By attaching to the APOC3, mRNA antisense oligonucleotides can stop it from producing the APOC3 protein, thus lowering its levels in the body [38]. The process through which antisense oligonucleotides work involves detailed steps. Initially, antisense oligonucleotides are made to match a sequence on the APOC3 mRNA. When administered, these antisense oligonucleotides pair with the target mRNA through base pairing, ensuring that only the mRNA of APOC3 is affected without influencing the expression of other genes or proteins [36-38]. This precision is essential for minimizing any unintended effects and maximizing treatment effectiveness. Once linked to the APOC3 mRNA, the antisense oligonucleotides complex brings in an enzyme known as RNase H. RNase H identifies the RNA DNA combination created by the binding of antisense oligonucleotides and mRNA and cuts the RNA strand [39,40]. This cutting leads to the breaking down of the APOC3 mRNA, halting its translation into the APOC3 protein [41]. By lowering the amounts of the APOC3 protein, antisense oligonucleotides are able to reduce its ability to hinder the functions of LPL and hepatic lipase, resulting in improved removal of triglycerides from the bloodstream.

Volanesorsen, a type of medication that targets APOC3 mRNA using antisense oligonucleotides, has proven effective in reducing triglyceride levels in individuals with familial chylomicronemia syndrome, a rare genetic condition characterized by extremely high levels of triglycerides. Clinical trials have shown that volanesorsen can lower plasma triglyceride levels by as much as 77%, suggesting its potential as a robust treatment for severe hypertriglyceridemia. The findings of two clinical trials, COMPASS and APPROACH, are summarized in Table 1 [42,43].

**Table 1:** Summary of key results from the COMPASS and APPROACH trials evaluating the safety and efficacy of volanesorsen in patients with severe hypertriglyceridemia.

	COMPASS trial [42]	APPROACH trial [43]
Study type	Phase 3, randomized, placebo-controlled, double-blind	Phase 3, randomized, placebo-controlled, double-blind
Population	Patients with multifactorial severe hypertriglyceridemia or familial chylomicronaemia syndrome	Patients with familial chylomicronemia syndrome
Number of patients	114 (76 volanesorsen group, 38 placebo group)	66 (33 volanesorsen group, 33 placebo group)
Triglyceride change (volanesorsen group)	71.2% reduction from baseline (mean absolute reduction of 869 mg/dL)	77% reduction from baseline (mean absolute reduction of 1712 mg/dL)
Triglyceride change (placebo group)	0.9% increase from baseline (mean absolute increase of 74 mg/dL)	18% increase from baseline (mean absolute increase of 92 mg/dL)
Adverse events (volanesorsen group)	Injection-site reactions (24% of injections), one case of platelet count <50,000/μL, one case of serum sickness	Injection-site reactions (20/33 patients), thrombocytopenia (15/33 patients had platelet counts <100,000/μL, two had <25,000/μL)
Adverse events (placebo group)	Injection-site reactions (0.2% of injections), five cases of acute pancreatitis	No injection-site reactions, no thrombocytopenia

The COMPASS trial included 114 participants who were randomly assigned to either receive volanesorsen (76 individuals) or a placebo (38 individuals). It demonstrated reductions in average plasma triglyceride levels with volanesorsen. The group receiving volanesorsen experienced a 71.2% decrease from baseline to three months, while the placebo group saw a 0.9% increase (p < 0.0001). This resulted in an absolute reduction of 869 mg/dL (9.82 mmol/L) in the volanesorsen group compared to a 74 mg/dL (0.83 mmol/L) increase in the placebo group (p < 0.0001). Notably, during the study period, all five cases of acute pancreatitis were reported among participants in the placebo group [42]. Volanesorsen was generally well-tolerated, with injection-site reactions being the most common adverse event. This study had several limitations including a short treatment period, a small sample size, and the enrollment of a predominantly White population.

The APPROACH trial was a phase 3 trial lasting 52 weeks and involving 66 patients with familial chylomicronemia syndrome conducted under double-blind conditions. Volanesorsen was found to significantly lower triglyceride levels. Patients who were given volanesorsen experienced an 84% drop in average plasma APOC3 levels (25.7 mg/dL) after three months, while the placebo group saw a 6.1% increase (1.9 mg/dL) (p < 0.001). Furthermore, volanesorsen resulted in a 77% decrease in average triglyceride levels (decrease of 1712 mg/dL or 19.3 mmol/L from baseline; 95% CI: 1330 to 2094 mg/dL), whereas those on placebo showed an 18% rise (average increase of 92.0 mg/dL or 1.0 mmol/L from baseline; 95% CI: 301.0 to 486 mg/dL) (p < 0.001). After three months, the percentage of participants with triglyceride levels below 750 mg/dL in the volanesorsen group was 77% compared with 10% in the placebo group. Thrombocytopenia (15/33) and injection-site reactions (20/33) were common adverse events reported in this study [43]. These studies lacked data on the long-term effects of lowering triglycerides such as major adverse cardiovascular events, stroke, or pancreatitis.

With regard to its mechanism of action, APOC3 affects metabolism through various pathways including inhibiting LPL activity. LPL functions to aid in the breakdown of triglycerides in chylomicrons and VLDL particles. The inhibition of LPL through the activity of APOC3 leads to the accumulation of these particles in the bloodstream. Additionally, APOC3 interferes with the liver's ability to uptake particle remnants, contributing further to increased triglyceride levels. Moreover, APOC3 boosts VLDL secretion from the liver, worsening hypertriglyceridemia. Collectively, these effects are crucial in contributing to plasma triglyceride levels [4,44,45].

APOC3 not only affects triglyceride metabolism but also has broader implications on overall metabolism. Elevated APOC3 levels contribute to increased insulin resistance, leading to a cycle that worsens hypertriglyceridemia and raises the risk of metabolic syndrome and type 2 diabetes [46]. Understanding how APOC3 interacts with insulin resistance can help researchers create specific interventions for these interconnected metabolic issues. Studying APOC3 and severe hypertriglyceridemia provides insights with significant treatment implications [47,48]. Current approaches for hypertriglyceridemia focus on lifestyle changes, like diet and exercise, as well as medications such as fibrates, omega-3 fatty acids, and statins. The American Heart Association (AHA) and the American College of Cardiology (ACC) stress the importance of these lifestyle modifications, recommending a diet that's low in saturated fats and refined carbs, increased exercise, and weight loss as key measures to control high triglyceride levels [49].

However, in cases of severe hypertriglyceridemia, these methods may not always be sufficient. According to guidelines from the Endocrine Society, patients with elevated triglyceride levels (≥500 mg/dL) may require more aggressive medication to lower their risk of pancreatitis [50]. Fibrates are highlighted as effective in reducing triglyceride levels and are commonly prescribed as a first-line treatment in such scenarios [51,52]. Various large-scale studies have explored treatments for hypertriglyceridemia. For instance, the Reduction of Events with Icosapent Ethyl-Intervention Trial (REDUCE-IT) revealed that high-dose icosapent ethyl, a type of eicosapentaenoic acid (EPA), significantly decreased cardiovascular events in patients with high triglycerides despite their being on statin therapy [53]. Another study, the ACCORD trial looked into how including fenofibrate alongside statin treatment affected patients with type 2 diabetes. The study revealed a decrease in cardiovascular incidents among patients with elevated triglyceride levels and low HDL cholesterol levels [54]. However, these methods often fall short in managing severe cases.

In summary, the link between APOC3 and severe hypertriglyceridemia is intricate and diverse. Genetic research highlights the role of APOC3 in triglyceride metabolism, while clinical trials demonstrate the potential benefits of targeting APOC3 for treating severe hypertriglyceridemia. Researchers can enhance the effectiveness of treatments and improve outcomes for patients by understanding how APOC3 impacts lipid metabolism.

Therapeutic implications

Exploring the role of APOC3 in managing lipid levels and its impact on hypertriglyceridemia offers new possibilities for treatment options. Addressing hypertriglyceridemia typically involves making lifestyle changes using medications as well as considering available and upcoming therapies that target APOC3. Making adjustments to one’s lifestyle is essential in the management of hypertriglyceridemia. Lifestyle changes include modifying diet by reducing the intake of carbs, sugars, and saturated fats while increasing omega-3 fatty acid consumption. Engaging in aerobic physical activity is also beneficial for improving lipid levels and overall metabolic health [55,56].

Medications are often required for individuals with hypertriglyceridemia. Common drugs like fibrates help lower levels by enhancing LPL activity. Omega 3 fatty acid supplements containing EPA and docosahexaenoic acid (DHA) are also used to reduce triglycerides. Statins, primarily used to lower LDL cholesterol, can offer significant relief in reducing triglycerides, especially when there is mixed dyslipidemia present. However, these treatments may not always suffice for cases of hypertriglyceridemia, underscoring the necessity for more targeted therapies [57,58].

New treatments focusing on APOC3 show promise as an approach to managing severe hypertriglyceridemia. Antisense oligonucleotides are created to inhibit protein production by targeting the specific mRNAs responsible for the individual proteins they are coded for. Volanesorsen, a medication that targets APOC3 mRNA using antisense oligonucleotides technology, has proven effective in lowering plasma triglyceride levels. Studies have shown that volanesorsen can reduce triglycerides by as much as 77% in individuals with familial chylomicronemia syndrome, a condition characterized by dangerously high triglyceride levels that come along with the risk of recurring pancreatitis. This significant decrease in triglycerides brings hope to patients who do not respond well to other triglyceride-lowering treatments [42,43].

The mechanism of action of volanesorsen is by binding to APOC3 mRNA, which stops the translation of the APOC3 mRNA into functional APOC3 protein. By decreasing levels of APOC3 protein, LPL is disinhibited, which allows for greater breakdown of triglycerides in chylomicrons and VLDL particles. This method emphasizes the potential of therapies focused on lowering APOC3 to target the cause of severe hypertriglyceridemia.

Apart from volanesorsen, there are other treatment strategies being explored that target APOC3. One of these strategies includes monoclonal antibodies designed to counteract the effects of the APOC3 protein [59]. These treatments could present an alternative to antisense oligonucleotides and potentially achieve similar benefits in reducing triglyceride levels while offering different pharmacokinetic and safety profiles.

The advancement of therapies targeting APOC3 shows potential for enhancing the management of severe hypertriglyceridemia. These treatments hold promise for achieving effective and lasting reductions in triglyceride levels compared to current therapies. This could lead to a reduction in the risk of issues like acute pancreatitis and heart diseases, leading to better results and increased quality of life for patients. However, the use of treatments that target APOC3 raises concerns about the safety and effectiveness of lowering APOC3 activity in the long term. The effects of the inhibition of APOC3 on overall lipid metabolism and possible unintended consequences should be thoroughly examined in clinical studies. Furthermore, it is crucial to define the criteria for selecting patients to determine who would benefit most from these new therapies.

In clinical practice, incorporating APOC3-targeted treatments into current care plans will require a personalized approach. Factors such as the severity of triglyceride levels, genetic predispositions, and existing health conditions must be taken into account. Ongoing studies and trials will play a role in refining these treatments and establishing their place in managing severe hypertriglyceridemia comprehensively.

Future directions

Most research conducted up to date has concentrated on particular genetic variations found in specific ethnic groups, yet there is still much to explore regarding the global genetic landscape of APOC3 mutations. Studying diverse populations can lead to the discovery of new mutations and their effects, offering fresh perspectives on the involvement of APOC3 in conditions like hypertriglyceridemia and other lipid disorders.

Future studies should focus on filling these research gaps by conducting term clinical trials and thorough genetic assessments. It is crucial to monitor the lasting effectiveness and safety of APOC3-targeted therapies through studies involving varied patient groups encompassing different genetic backgrounds for broad applicability. Secondly, the long-term effects of lowering triglycerides should be studied. Outcomes like stroke, major adverse cardiovascular events, or mortality need to be studied and linked to the effects of triglyceride-lowering therapy.

Delving into the mechanisms governing APOC3's interactions with other pathways related to lipid metabolism will demand sophisticated biochemical and genetic methodologies. Research employing cutting-edge tools like CRISPR gene editing, RNA sequencing, and proteomics could offer insights into these intricate connections. Furthermore, utilizing animal models with APOC3 mutations can provide controlled environments for studying the physiological impacts of these mutations, shedding light on their contributions to lipid metabolism disorders.

Exploring potential other methods for inhibiting APOC3 shows a lot of potential. Apart from using oligonucleotides and antibodies, we can look into other approaches as well. Developing molecules that control APOC3 function or disrupt its interactions with other proteins could offer new treatment options. Personalized medicine strategies could optimize the use of APOC3-targeted treatments. By customizing therapies based on genetic and metabolic traits, healthcare professionals can maximize effectiveness while minimizing side effects. Identifying biomarkers that forecast responses to APOC3-focused therapies could assist in selecting patients, monitoring treatment progress, and improving outcomes.

## Conclusions

Severe hypertriglyceridemia is a condition with significant health implications, such as an increased risk of heart disease and acute pancreatitis. APOC3 plays a role in controlling triglyceride levels in the body through various mechanisms. Certain genetic mutations in APOC3 can lead to lower triglyceride levels and reduce the likelihood of heart disease. This has spurred the development of treatments targeting APOC3 to manage hypertriglyceridemia. The treatment landscape for hypertriglyceridemia is changing, with new therapies like antisense oligonucleotides showing promise. Volanesorsen, an antisense oligonucleotide targeting APOC3, has proven effective in lowering triglyceride levels in patients with familial chylomicronemia syndrome. These advancements suggest that APOC3-targeted therapies could offer more management options for hypertriglyceridemia than traditional methods.

However, there are still challenges and opportunities for research. The safety and effectiveness of APOC3-targeted therapies need assessment, especially regarding their impact on overall lipid metabolism and potential side effects. Understanding how APOC3 interacts with metabolic pathways and the genetic variations of APOC3 in populations will help develop more personalized treatment strategies. In the future, it is important for research to focus on filling these gaps by conducting term trials utilizing advanced biochemical and genetic studies and fostering collaboration across various fields. Exploring treatment approaches such as molecule inhibitors, monoclonal antibodies, and gene editing technologies can help broaden the range of available therapies for severe hypertriglyceridemia. By deepening our knowledge of APOC3 and harnessing state-of-the-art methods, we can make strides toward personalized treatments for severe hypertriglyceridemia, ultimately improving the quality of life for individuals affected by this disorder.

## References

[1] Yuan G, Al-Shali KZ, Hegele RA (2007). Hypertriglyceridemia: its etiology, effects and treatment. CMAJ.

[2] Berglund L, Brunzell JD, Goldberg AC, Goldberg IJ, Sacks F, Murad MH, Stalenhoef AF (2012). Evaluation and treatment of hypertriglyceridemia: an Endocrine Society clinical practice guideline. J Clin Endocrinol Metab.

[3] Parhofer KG, Laufs U (2019). The diagnosis and treatment of hypertriglyceridemia. Dtsch Arztebl Int.

[4] Borén J, Packard CJ, Taskinen MR (2020). The roles of ApoC-III on the metabolism of triglyceride-rich lipoproteins in humans. Front Endocrinol (Lausanne).

[5] Larsson M, Vorrsjö E, Talmud P, Lookene A, Olivecrona G (2013). Apolipoproteins C-I and C-III inhibit lipoprotein lipase activity by displacement of the enzyme from lipid droplets. J Biol Chem.

[6] Dib I, Khalil A, Chouaib R, El-Makhour Y, Noureddine H (2021). Apolipoprotein C-III and cardiovascular diseases: when genetics meet molecular pathologies. Mol Biol Rep.

[7] Goyal S, Tanigawa Y, Zhang W (2021). APOC3 genetic variation, serum triglycerides, and risk of coronary artery disease in Asian Indians, Europeans, and other ethnic groups. Lipids Health Dis.

[8] Hernandez P, Passi N, Modarressi T (2021). Clinical management of hypertriglyceridemia in the prevention of cardiovascular disease and pancreatitis. Curr Atheroscler Rep.

[9] Fan W, Philip S, Granowitz C, Toth PP, Wong ND (2020). Prevalence of US adults with triglycerides ≥ 150 mg/dl: NHANES 2007-2014. Cardiol Ther.

[10] Carrasquilla GD, Christiansen MR, Kilpeläinen TO (2021). The genetic basis of hypertriglyceridemia. Curr Atheroscler Rep.

[11] Luna-Castillo KP, Olivares-Ochoa XC, Hernández-Ruiz RG (2022). The effect of dietary interventions on hypertriglyceridemia: from public health to molecular nutrition evidence. Nutrients.

[12] Denisenko YK, Kytikova OY, Novgorodtseva TP, Antonyuk MV, Gvozdenko TA, Kantur TA (2020). Lipid-induced mechanisms of metabolic syndrome. J Obes.

[13] Halpern A, Mancini MC, Magalhães ME (2010). Metabolic syndrome, dyslipidemia, hypertension and type 2 diabetes in youth: from diagnosis to treatment. Diabetol Metab Syndr.

[14] Subramanian S, Chait A (2012). Hypertriglyceridemia secondary to obesity and diabetes. Biochim Biophys Acta.

[15] Gugliucci A (2023). Sugar and dyslipidemia: a double-hit, perfect storm. J Clin Med.

[16] Rygiel K (2018). Hypertriglyceridemia - common causes, prevention and treatment strategies. Curr Cardiol Rev.

[17] Mavromati M, Jornayvaz FR (2021). Hypothyroidism-associated dyslipidemia: potential molecular mechanisms leading to NAFLD. Int J Mol Sci.

[18] Toms TE, Panoulas VF, Kitas GD (2011). Dyslipidaemia in rheumatological autoimmune diseases. Open Cardiovasc Med J.

[19] Okazaki H, Gotoda T, Ogura M (2021). Current diagnosis and management of primary chylomicronemia. J Atheroscler Thromb.

[20] Taskinen MR, Borén J (2016). Why Is apolipoprotein CIII emerging as a novel therapeutic target to reduce the burden of cardiovascular disease?. Curr Atheroscler Rep.

[21] Giammanco A, Spina R, Cefalù AB, Averna M (2023). APOC-III: a gatekeeper in controlling triglyceride metabolism. Curr Atheroscler Rep.

[22] Mead JR, Irvine SA, Ramji DP (2002). Lipoprotein lipase: structure, function, regulation, and role in disease. J Mol Med (Berl).

[23] Das P, Ingole N (2023). Lipoproteins and their effects on the cardiovascular system. Cureus.

[24] Chatterjee C, Sparks DL (2011). Hepatic lipase, high density lipoproteins, and hypertriglyceridemia. Am J Pathol.

[25] Ormazabal V, Nair S, Elfeky O, Aguayo C, Salomon C, Zuñiga FA (2018). Association between insulin resistance and the development of cardiovascular disease. Cardiovasc Diabetol.

[26] Florez H, Mendez A, Casanova-Romero P, Larreal-Urdaneta C, Castillo-Florez S, Lee D, Goldberg R (2006). Increased apolipoprotein C-III levels associated with insulin resistance contribute to dyslipidemia in normoglycemic and diabetic subjects from a triethnic population. Atherosclerosis.

[27] Paiva AA, Raposo HF, Wanschel AC, Nardelli TR, Oliveira HC (2017). Apolipoprotein CIII overexpression-induced hypertriglyceridemia increases nonalcoholic fatty liver disease in association with inflammation and cell death. Oxid Med Cell Longev.

[28] Lee HY, Birkenfeld AL, Jornayvaz FR (2011). Apolipoprotein CIII overexpressing mice are predisposed to diet-induced hepatic steatosis and hepatic insulin resistance. Hepatology.

[29] Åvall K, Ali Y, Leibiger IB (2015). Apolipoprotein CIII links islet insulin resistance to β-cell failure in diabetes. Proc Natl Acad Sci U S A.

[30] Crosby J, Peloso GM, Auer PL (2014). Loss-of-function mutations in APOC3, triglycerides, and coronary disease. N Engl J Med.

[31] Jørgensen AB, Frikke-Schmidt R, Nordestgaard BG, Tybjærg-Hansen A (2014). Loss-of-function mutations in APOC3 and risk of ischemic vascular disease. N Engl J Med.

[32] Schmitz J, Gouni-Berthold I (2018). APOC-III antisense oligonucleotides: a new option for the treatment of hypertriglyceridemia. Curr Med Chem.

[33] Qamar A, Libby P, Bhatt DL (2019). Targeting RNA to lower triglycerides: long strides from short molecules. Eur Heart J.

[34] Kohan AB (2015). Apolipoprotein C-III: a potent modulator of hypertriglyceridemia and cardiovascular disease. Curr Opin Endocrinol Diabetes Obes.

[35] Taskinen MR, Packard CJ, Borén J (2019). Emerging evidence that ApoC-III inhibitors provide novel options to reduce the residual CVD. Curr Atheroscler Rep.

[36] Masson W, Lobo M, Nogueira JP, Corral P, Barbagelata L, Siniawski D (2024). Inhibitors of apolipoprotein C3, triglyceride levels, and risk of pancreatitis: a systematic review and meta-analysis. Rev Endocr Metab Disord.

[37] Collotta D, Bertocchi I, Chiapello E, Collino M (2023). Antisense oligonucleotides: a novel frontier in pharmacological strategy. Front Pharmacol.

[38] Alexander VJ, Xia S, Hurh E (2019). N-acetyl galactosamine-conjugated antisense drug to APOC3 mRNA, triglycerides and atherogenic lipoprotein levels. Eur Heart J.

[39] Liang XH, Sun H, Nichols JG, Crooke ST (2017). RNase H1-dependent antisense oligonucleotides are robustly active in directing RNA cleavage in both the cytoplasm and the nucleus. Mol Ther.

[40] Kole R, Krainer AR, Altman S (2012). RNA therapeutics: beyond RNA interference and antisense oligonucleotides. Nat Rev Drug Discov.

[41] Chery J (2016). RNA therapeutics: RNAi and antisense mechanisms and clinical applications. Postdoc J.

[42] Gouni-Berthold I, Alexander VJ, Yang Q (2021). Efficacy and safety of volanesorsen in patients with multifactorial chylomicronaemia (COMPASS): a multicentre, double-blind, randomised, placebo-controlled, phase 3 trial. Lancet Diabetes Endocrinol.

[43] Witztum JL, Gaudet D, Freedman SD (2019). Volanesorsen and triglyceride levels in familial chylomicronemia syndrome. N Engl J Med.

[44] Packard CJ, Boren J, Taskinen MR (2020). Causes and consequences of hypertriglyceridemia. Front Endocrinol (Lausanne).

[45] Gugliucci A (2023). Triglyceride-rich lipoprotein metabolism: key regulators of their flux. J Clin Med.

[46] Buckner T, Shao B, Eckel RH, Heinecke JW, Bornfeldt KE, Snell-Bergeon J (2021). Association of apolipoprotein C3 with insulin resistance and coronary artery calcium in patients with type 1 diabetes. J Clin Lipidol.

[47] Martín-González C, Martín-Folgueras T, Quevedo-Abeledo JC (2022). Apolipoprotein C-III is linked to the insulin resistance and beta-cell dysfunction that are present in rheumatoid arthritis. Arthritis Res Ther.

[48] Luciani L, Pedrelli M, Parini P (2024). Modification of lipoprotein metabolism and function driving atherogenesis in diabetes. Atherosclerosis.

[49] Grundy SM, Stone NJ, Bailey AL (2019). 2018 AHA/ACC/AACVPR/AAPA/ABC/ACPM/ADA/AGS/APhA/ASPC/NLA/PCNA guideline on the management of blood cholesterol: a report of the American College of Cardiology/American Heart Association Task Force on Clinical Practice Guidelines. Circulation.

[50] Newman CB, Blaha MJ, Boord JB (2020). Lipid management in patients with endocrine disorders: an endocrine society clinical practice guideline. J Clin Endocrinol Metab.

[51] Tenenbaum A, Fisman EZ (2012). Fibrates are an essential part of modern anti-dyslipidemic arsenal: spotlight on atherogenic dyslipidemia and residual risk reduction. Cardiovasc Diabetol.

[52] Berberich AJ, Hegele RA (2022). A modern approach to dyslipidemia. Endocr Rev.

[53] Bhatt DL, Steg PG, Miller M (2019). Cardiovascular risk reduction with icosapent ethyl for hypertriglyceridemia. N Engl J Med.

[54] Elam M, Lovato L, Ginsberg H (2011). The ACCORD-Lipid study: implications for treatment of dyslipidemia in type 2 diabetes mellitus. Clin Lipidol.

[55] Ohni M (2013). Lifestyle modifications for treatment of hypertriglyceridemia [Article in Japanese]. Nihon Rinsho.

[56] Byrne A, Makadia S, Sutherland A, Miller M (2017). Optimizing non-pharmacologic management of hypertriglyceridemia. Arch Med Res.

[57] Moon JH, Kim K, Choi SH (2022). Lipoprotein lipase: is it a magic target for the treatment of hypertriglyceridemia. Endocrinol Metab (Seoul).

[58] Ki YJ, Han SJ, Cha TJ (2022). A multi-center, prospective observational study to investigate the safety, compliance, and efficacy of omethyl QTlet soft capsule. J Clin Med.

[59] Hermel M, Lieberman M, Slipczuk L, Rana JS, Virani SS (2023). Monoclonal antibodies, gene silencing and gene editing (CRISPR) therapies for the treatment of hyperlipidemia-the future is here. Pharmaceutics.

